# Supplementary figures and images for: The Route of HIV Escape from Immune Response Targeting Multiple Sites Is Determined by the Cost-Benefit Tradeoff of Escape Mutations
Source: PLoS Comput Biol. 2014 Oct 30;10(10):e1003878. doi: 10.1371/journal.pcbi.1003878 (PMC4214571; doi:10.1371/journal.pcbi.1003878)

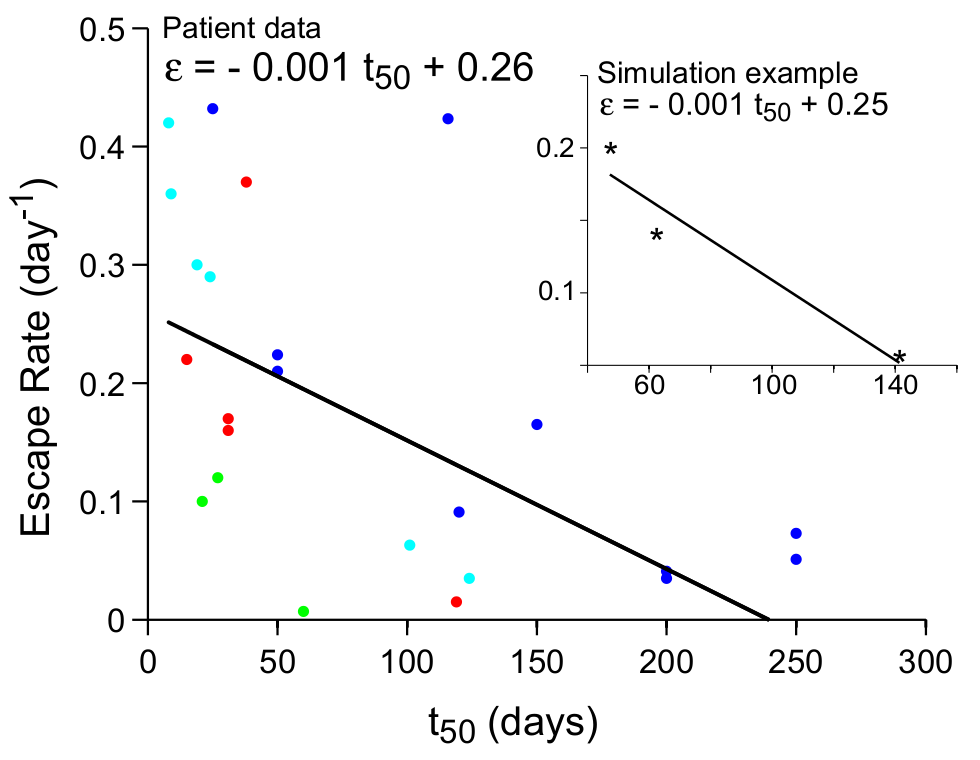

Supplement: Figure S1 — Escape rate and are negatively correlated in two experimental studies. The frequency of a mutated epitope for epitopes over time is fit to the curve , which describes deterministic selection on a single site with selection coefficient , in order to determine parameters and . Colored dots show data from a single patient studied in [11] (blue) and multiple patients studied in [30] CH40 (red), CH58 (green), CH77 (cyan). Inset: Simulation example showing the correlation between escape rate, , and the time that the mutation spreads to 50% of the population of infected cells, denoted . Parameters and are found for the three escape mutations shown in Figure 1B that occur in the first 200 days post infection. Thus, variation in recognition and fitness losses across many epitopes successfully reproduces this feature of escape dynamics. (TIF) [file pcbi.1003878.s001.tif]

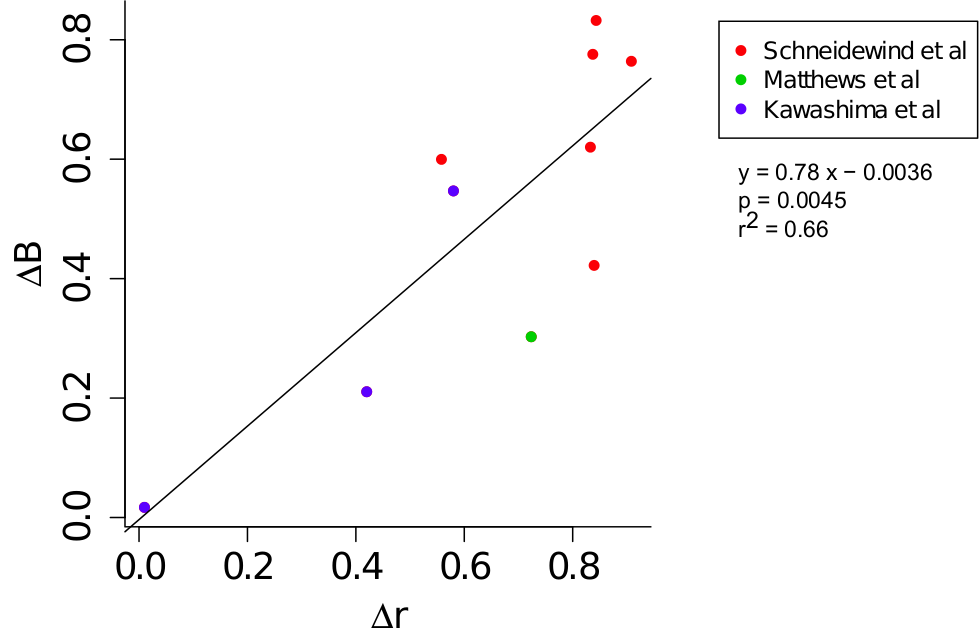

Supplement: Figure S2 — Estimating the relationship between and approximately from three published experiments. Our model contains one parameter for CTL recognition loss caused by a mutation, . In order to compare model predictions with data from Mostowy et al [26], where HLA binding impairment caused by mutations was considered rather than overall CTL recognition loss, we sought to compare HLA binding impairment with overall CTL recognition loss. By combine data from three references, Schneidewind et al [27], Kawashima et al [28] and Matthews et al [29], we were able to demonstrate a strong correlation between the two parameters. This justifies our comparison of our model predictions with data from [26]. (TIF) [file pcbi.1003878.s002.tif]

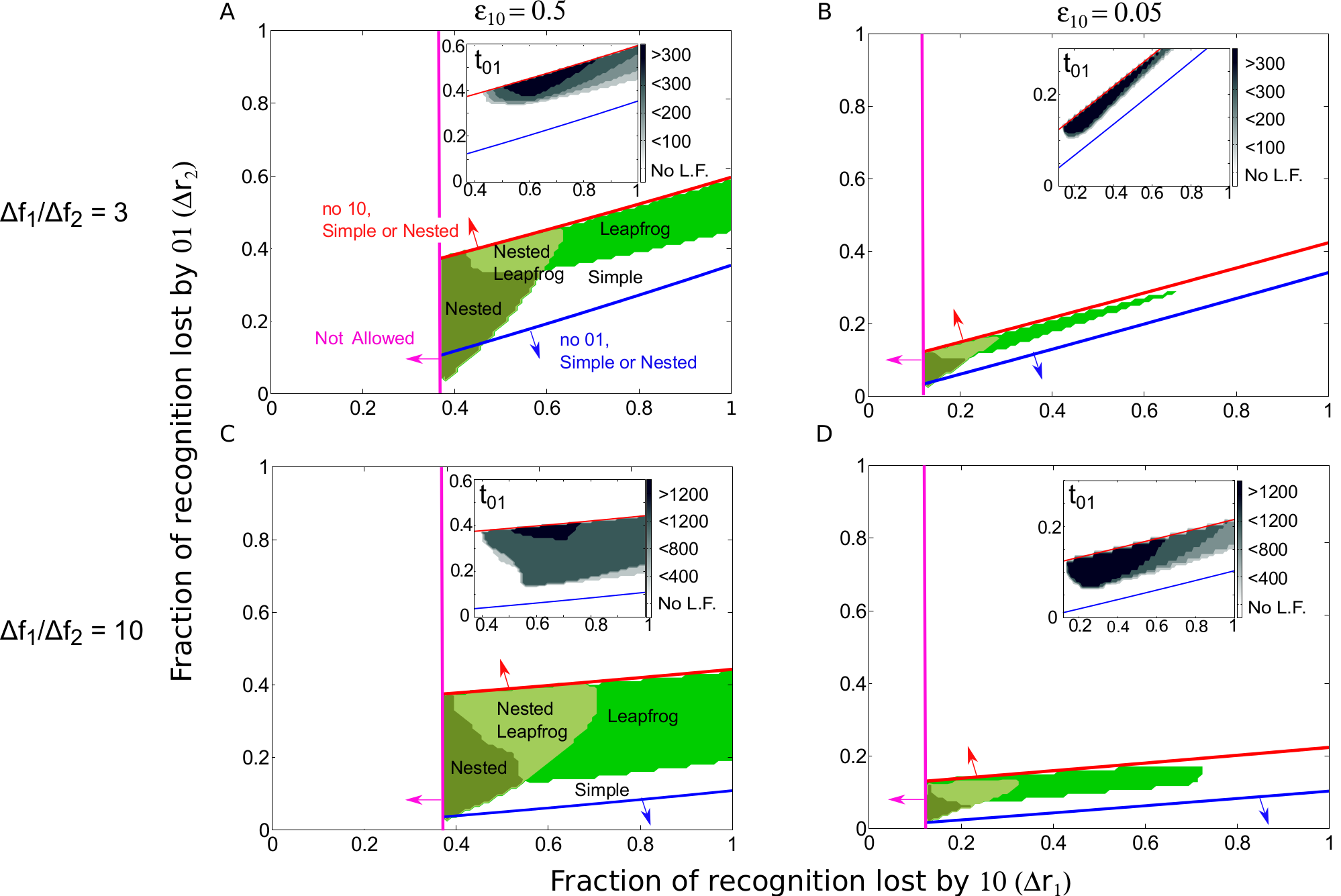

Supplement: Figure S3 — CTL lifetime changes the frequency with which intra-epitope escape patterns are observed. Here we show a modified version of Figure 5 for the case when CTL are long-lived, . Using simulation of the model (Figure 1A, Equations 6 to 8) with two sites per epitope, , the pattern of escape is calculated for a range of recognition and fitness losses. The pattern that is obtained is plotted as a function of the parameters of recognition loss at the first and second site ( and , respectively). In each panel, certain parameters are fixed in order to focus on the effect of recognition loss. Fixed parameters are: the escape rate of the first haplotype and the number of targeted epitopes: , (A,C) which correspond to early infection and , (B,D) which correspond to chronic infection. Fitness costs are chosen such that the second site is less costly than the first: equal to 3 (A,B) or much less costly than the first, (C,D). Other parameters given in Table 1. Equations S6 (red line) and S9 (blue line) determine the region where the leapfrog pattern can be observed. Regions that require are not allowed by definition (magenta line). The shaded regions between these three lines correspond to regions of parameter space where both sites escape. The corresponding patterns are: “leapfrog” (, Figure 4C), “nested” (, Figure 4E), “nested leapfrog” (). Observation of the leapfrog pattern in an epitope tightly constrains the fraction of CTL recognition loss conferred by sites in an epitope. The inset shows the length of time during which haplotype 01 is dominant in the escaping epitope. When CTLs are long-lived, the leapfrog pattern (Figure 4C) is observed more often, since the haplotype 01 has time to grow to dominate the population. The death rate of CTL is , ten times smaller than the value used in Figure 5. The difference between the predicted leapfrog region as determined by Equations S6 (red line) and S9 (blue line) and the shaded region where leapfrog is actually observed is lessened fo [file pcbi.1003878.s003.tif]
