# Supplementary material for: The Route of HIV Escape from Immune Response Targeting Multiple Sites Is Determined by the Cost-Benefit Tradeoff of Escape Mutations
Source: PLoS Comput Biol. 2014 Oct 30;10(10):e1003878. doi: 10.1371/journal.pcbi.1003878 (PMC4214571; doi:10.1371/journal.pcbi.1003878)
Supplement: Text S1 — Steady state and the escape rate. (PDF) [file pcbi.1003878.s004.pdf]

S1 Steady state and the escape rate

We describe the approach to the steady state that occurs for the system described in Equations 6 to 8. Initially the population is uniform in the transmitted strain (I_{trans}). Expansion of the population of infected cells causes all n CTL clones to expand. The expansion of CTL, in turn, reduces infected cell levels until the expansion of CTL is balanced by their death (first and second phases in Figure 1B). The system approaches a steady state that can be obtained from Equation 6 - 8 with all $r_{\text{ij}} = 1$ and $f_{\text{i}} = 1$.

$$T^{\text{ss}} = \frac{\lambda}{d_T + \beta I^{\text{ss}}} \tag{S1}$$

$$I^{\text{ss}} \approx \frac{d_E h}{c}, \quad d_E \ll c \tag{S2}$$

$$E_{\text{tot}} \equiv \frac{\beta T^{\text{ss}} - d_I}{\kappa}, \sum\nolimits_j E_j^{\text{ss}} = E_{\text{tot}} \tag{S3}$$

The level of infected cells in steady state is proportional to the inverse avidity of the CTL clones, h. In this work, we consider n equal-avidity CTL clones with equal precursor frequencies. The steady state is an indifferent equilibrium, in which each CTL clone comprises on the order of $1/n$ of the CTL population, E_{tot}. Differences in precursor frequencies, as well as stochastic fluctuations in steady state can result in different levels of CTL in steady state. However, our qualitative conclusions are independent of modest differences in initial CTL levels (see *Discussion*).

We can derive the escape rate of the first escape mutant, Equation 1 as follows. When the population is uniform in the transmitted strain, a mutated strain with a single mutation in epitope one (I_i) has the growth rate given by Equation 7, with fitness and recognition reduced by factors $\Delta f_i = 1 - e^{-s_i}$ and $\Delta r_i = 1 - e^{-r_i}$, respectively.

$$\frac{dI_i}{dt} = \left[\beta T^{\text{ss}}(1 - \Delta f_i) - d_I - \kappa\left(\sum_j E_j^{\text{ss}} - E_1^{\text{ss}}\Delta r_i\right)\right] I_i \tag{S4}$$

Assuming cell populations have reached steady state (Equations S1-S3), we can substitute Equation S3 and $E_1 = kE_{\text{tot}}/n_1$, where $1/n_1$ is the fraction of CTL population occupied by CTL clone 1, into Equation S4.
