# Supplementary material for: The Route of HIV Escape from Immune Response Targeting Multiple Sites Is Determined by the Cost-Benefit Tradeoff of Escape Mutations
Source: PLoS Comput Biol. 2014 Oct 30;10(10):e1003878. doi: 10.1371/journal.pcbi.1003878 (PMC4214571; doi:10.1371/journal.pcbi.1003878)
Supplement: Text S2 — Escape causes contraction of CTL clones. (PDF) [file pcbi.1003878.s005.pdf]

S2 Escape causes contraction of CTL clones

Here we derive the behavior of a CTL clone after an escape mutation in the corresponding epitope has occurred. We define CTL thresholds (I_j^{th} for the CTL clone recognizing epitope j), to be the level of infected cells that will keep the CTL clone in steady state. As an escape mutation in an epitope has spread to the majority of the population, CTLs to that epitope recognize infected cells less efficiently. This can be expressed as a new threshold for that clone, since number of infected cells that would be necessary to maintain the clone in steady state is elevated. CTL thresholds rise simultaneously with the emergence of escape mutations, plateauing when escape is complete. For a reduction in recognition relative to the transmitted strain of Δr_j, the plateau given by $I_j^{\text{th}} = I^{\text{ss}}/(1 - \Delta r_j)$.

Before escape mutations rise to high frequencies in the population, all CTL clones have the same threshold, $I_j^{\text{th}} = I^{\text{ss}}$. CTL clones that target epitopes unescaped epitopes recognize all infected cells in the population equally, regardless of sequence, and thus maintain their original, lower threshold. Therefore, as long as there is at least one epitope that has not escaped, the steady state level of infected cells will be held fixed by the CTL populations targeting conserved epitopes (Figure 1B). CTLs to escaped epitopes receive less stimulation from infected cells due to raised thresholds and decline according to:

$$E_j(t) = \frac{E_{\text{tot}}}{n} e^{-d_E \Delta r_j t} \tag{S5}$$

where we have used Equation 8 and the strong inequality $\frac{d_E}{c}(1 - \Delta r_j) \ll 1$ (Table 1).

Thus, CTL clones to escaped epitopes decay even though recognition losses are partial ($\Delta r_j > 0$), due to the presence of other CTL clones targeting unescaped epitopes (Figure 1B). As mentioned previously, the contraction of CTLs eventually causes reversion to the transmitted haplotype in the absence of compensatory mutations, see *Discussion*.

The size of the total CTL population (E_{tot}) responds to changes in the replication rate of the virus βT^{ss} (Equation S3). Throughout this work we consider small fitness costs ($\Delta f_j \sim 0$), such that the steady state number of target cells (Equation S1) and in the total CTL population, E_{tot} change very little. With these assumptions, the predictions of our model are consistent with the experimental observation that although the composition of the CTL population changes, the overall levels of CTL and target cells change slowly during chronic infection.

Upon reversion of an escape mutant, CTLs targeting that epitope cease to contract but do not re-expand. This is due to the viral load being maintained at the initial steady state level by other CTL clones that continue to target the un-escaped epitopes. In order to induce proliferation of these clones, the viral load would need to rise above the initial steady state level.
