# Supplementary material for: The Route of HIV Escape from Immune Response Targeting Multiple Sites Is Determined by the Cost-Benefit Tradeoff of Escape Mutations
Source: PLoS Comput Biol. 2014 Oct 30;10(10):e1003878. doi: 10.1371/journal.pcbi.1003878 (PMC4214571; doi:10.1371/journal.pcbi.1003878)
Supplement: Text S3 — General conditions for observing the leapfrog pattern of escape. (PDF) [file pcbi.1003878.s006.pdf]

S3 General conditions for observing the leapfrog pattern of escape

The most interesting pattern of intra-epitope escape, the leapfrog pattern, is characterized by a switch in the dominant single-mutant haplotype in an epitope. For example, in an epitope with two sites, the sequence of haplotypes observed in an epitope is 00 $\rightarrow$ 10 $\rightarrow$ 01, rather than the simple escape pattern 00 $\rightarrow$ 10 or the nested pattern 00 $\rightarrow$ 10 $\rightarrow$ 11, the later of which would be predicted if both sites were advantageous (i.e. have $\epsilon > 0$) and selection pressure were constant. Time-dependent CTL selection pressure can produce the leapfrog pattern. Here, we derive conditions on the fitness and recognition losses of both epitope sites that are necessary for the leapfrog pattern to occur. Without loss of generality, we consider the case where the initial escape rate of haplotype 10 in epitope one (ϵ_{10}) is higher than the initial escape rate of haplotype 01 (ϵ_{01}). Comparing initial escape rates when CTL are in steady state this condition reads:

$$\frac{\Delta r_1}{n} - \Delta f_1 > \frac{\Delta r_2}{n} - \Delta f_2 \tag{S6}$$

where we have used Equation 1 with the additional assumption that $d_I \ll kE_{\text{tot}}$, i.e. that the majority of infected cells are killed by CTL rather than by viral cytopathicity. We will use Equations 2 - 4 to determine conditions on the parameters of fitness and recognition losses under which the haplotype with the largest escape rate switches from 10 to 01.

In order for the leapfrog pattern to occur, haplotype 01 must gain the advantage over both haplotype 10 and the transmitted haplotypes, before the transmitted haplotype regains the advantage over the first. The CTL level at which haplotype 00 gains the advantage over 10 must be compared with the CTL level at which haplotype 01 gains the advantage over 10. We assume that haplotype 11 does not reach appreciable frequency before 01 sweeps, and neglect changes in the steady state level of target cells. Both are good approximations as long as the fitness costs at both epitope sites are small $\Delta f_1, \Delta f_2 \ll 1$, which we assume throughout this work. When the right-hand sides of Equations 2 and 3 are equal, haplotype 01 has the same selective advantage as haplotype 10. The fraction of the total CTL population made up by E_j at which this occurs is called $1/n_{\text{switch}}$, given by:

$$\frac{1}{n_{\text{switch}}} = \frac{\Delta f_1 - \Delta f_2}{\Delta r_1 - \Delta r_2} \tag{S7}$$

Haplotype 00 also has a lower intrinsic fitness cost than 10, and will begin to grow once $\epsilon_{10} = 0$. Reversion of haplotype 10 will begin once E_j has reached fraction $1/n_{\text{rev}}$, when Equation 2 is equal to zero:

$$\frac{1}{n_{\text{rev}}} = \frac{\Delta f_1}{\Delta r_1} \tag{S8}$$

The leapfrog pattern can be observed if and only if the switch occurs before the reversion of haplotype 10:

$$\frac{1}{n_{\text{switch}}} > \frac{1}{n_{\text{rev}}} \tag{S9}$$

Equations S6 and S9 state the conditions under which leapfrog pattern can be observed. These conditions can only be met if site 1 has both a greater fitness and recognition loss than site 2, which is given by Equation 5.
