# Supplementary material for: The Route of HIV Escape from Immune Response Targeting Multiple Sites Is Determined by the Cost-Benefit Tradeoff of Escape Mutations
Source: PLoS Comput Biol. 2014 Oct 30;10(10):e1003878. doi: 10.1371/journal.pcbi.1003878 (PMC4214571; doi:10.1371/journal.pcbi.1003878)
Supplement: Text S4 — Simulation: Leapfrog pattern can occur in a broad range of fitness and recognition losses. (PDF) [file pcbi.1003878.s007.pdf]

S4 Simulation: Leapfrog pattern can occur in a broad range of fitness and recognition losses

The model predicts that when fitness costs are small, partial recognition loss is the major determinant of which pattern of escape (simple, leapfrog, nested) is observed in an epitope. We use simulation to determine which pattern of escape is observed as a function of the fraction of recognition lost at either site (Figure 5). In order to do this, we hold constant the number of epitopes, n, the escape rate for the first haplotype, ϵ_{10}, and the ratio of the fitness costs in the two epitope sites, $\Delta f_1/\Delta f_2$. Two escape rates are shown that span the range of escape rates observed in data (Figure S1). For large escape rates, large values of Δr_1 are required, and a broad range of Δr_2 can produce the leapfrog pattern. Smaller values of Δr_1 produce smaller escape rates, and leapfrog is observed in a very narrow range of small Δr_2. The region where haplotype 11 is observed as a short intermediate between haplotypes 10 and 01 is labeled "nested leapfrog" in Figure 4.

The time interval for which a given haplotype dominates the population will depend on the parameters of loss and recognition at epitope sites, Δf_k and Δr_k. In [30], the 7/18 epitopes that display the leapfrog pattern show that the first haplotype is short lived compared to the second, and the transmitted epitope remains at low frequencies after it initially declines. Furthermore, reversion to the transmitted epitope is not always observed, possibly because the fitness cost incurred by the escape mutation is very small, or is compensated before the escape mutation begins to revert. The lifetimes of the two haplotypes thus provide estimates of the time interval during which compensation must occur. The absolute lifetime of the second haplotype (t_{01}) is therefore of interest, since it represents the amount of time during which the compensatory mutations are obtained that ameliorate this cost (inset of Figure 5). The fitness cost of the second haplotype is the major determinant of the lifetime, and for small fitness costs reversion to the transmitted epitope can take many years (Figure 5C,D).

Equations S6 and S9, as well as the condition that the fitness cost of site one is positive $\Delta f_1 > 0$, determine the region of parameter space where haplotype 01 gains the advantage over 10, and the leapfrog pattern can be observed. However, whether the second haplotype has enough time, once it becomes advantageous, to grow to dominate the population before starting to revert depends on the decay rate of CTL. If CTL are very long-lived in the absence of antigen, the lifetimes of the mutant haplotypes expand, as does the region of parameter space where leapfrog is observed (Figure S3).
