# Supplementary material for: The Route of HIV Escape from Immune Response Targeting Multiple Sites Is Determined by the Cost-Benefit Tradeoff of Escape Mutations
Source: PLoS Comput Biol. 2014 Oct 30;10(10):e1003878. doi: 10.1371/journal.pcbi.1003878 (PMC4214571; doi:10.1371/journal.pcbi.1003878)
Supplement: Text S5 — Finding the relationship between and HLA binding loss from three different experiments. (PDF) [file pcbi.1003878.s008.pdf]

S5 Finding the relationship between Δr and HLA binding loss from three different experiments

Finding Δr from dynamics of virus growth in the presence of CTL

In Schneidewind et al Figure 4C [27] the rate of virus growth in the presence of CTL was compared with the rate of virus growth without CTL for several mutations in the KK10 epitopes of Gag. We compare the growth of GFP_+ cells in the presence and absence of CTL to find the killing rate of CTL, kE, for both wild type and mutant virus. Then, by comparing the values of kE for wild type and mutant virus we can find the fractional loss of CTL recognition incurred by the mutant virus Δr. We start with the basic equation for number of GFP_+ over time:

$$GFP_+(t) = GFP_+(0)e^{(\beta - kE)t} \tag{S10}$$

Where β is the basic resplication rate of the virus and kE gives the CTL killing rate of infected cells. For each mutant, we find the value of GFP_+ at day 7 with and without CTL ($GFP_+CTL_+(7)$, $GFP_+CTL_-(7)$, respectively). We solve for kE:

$$kE = \frac{1}{7}\left[\log_e \frac{GFP_+CTL_-(7)}{GFP_+(0)} - \log_e \frac{GFP_+CTL_+(7)}{GFP_+(0)}\right] = \frac{1}{7}\log_e \frac{GFP_+CTL_-(7)}{GFP_+CTL_+(7)} \tag{S11}$$

where $GFP_+(0)$ was the initial value which cancels out. Comparing the CTL killing rate of wild type, kE_w, with the CTL killing rate of the mutant, kE_m we obtain:

$$\Delta r = 1 - \frac{(kE)_m}{(kE)_w} \tag{S12}$$

Fractional loss of HLA-binding (ΔB) from competition binding assay $IC50$ values

Using values from Table 1 in [27] we find the binding impairment of the mutant peptide, ΔB, as defined [26] (here we use the absolute criterion):

$$\Delta B = \frac{1}{5 \cdot 10^4}\log_e\left(\frac{IC50_m}{IC50_w}\right) \tag{S13}$$

ΔB and Δr from measurements of CTL activity at varying CTL concentration

When viral dynamics over time are not given, we must use a different method to find Δr. Both Kawashima et al [28] and Matthews et al [29] measure loss of HLA binding and loss of CTL function (either specific lysis or INF-γ secretion) for the same epitopes. First, we will use data from a third study by Tomiyama et al [71] to find a relationship between the concentration for 50% maximal binding (BL50) and the concentration for 50% maximal lysis (LL50). This study measured the ability of HLA-B*5101 restricted CTLs to recognize epitopes in several genes. We find the best linear fit, converting all units to nM, in order to find a peptide concentration that is relevant to both binding and lysis.

Next, we can find BL50 by fitting the relative mean fluorescence intensity (MFI) versus peptide concentration in the binding assay shown in Figure 1G in Kawashima et al [28] and Figure 6A in Matthews et al [29], using the following interpolation function:

$$y = \frac{[bottom - (top - bottom)]}{\left[1 + \left(\frac{\log(\text{Peptide Concentration})}{\text{BL50}}\right)\right]^{slope}} \tag{S14}$$

The function has four fitting parameters: *top* and *bottom* set the maximum and minimum values, BL50 gives the value of the function at half maximum and *slope* sets the shape of the curve.

Finally, we fit Equation S14 to the specific lysis % vs. peptide concentration as shown in Figure 1F in Kawashima et al [28] and the INF-γ SFC count in Figure 5B in Matthews et al [29]. We then determine the lysis value or INF-γ SFC count at the peptide concentration corresponding to BL50 (LL50atBL50). This is done in order to ensure that the peptide concentration that we consider is relevant to both binding and lysis. Finally, we find Δr using Equation S12, where for these two experiments kE found for the wild type and mutant epitope by the lysis % or INF-γ SFC count at the peptide concentration LL50atBL50 found from the above fitting. We obtain Table S1 and Table S2.When estimates from the three experiments are combined, we obtain a strong correlation between Δr and ΔB, $\Delta B = 0.78\Delta r - 0.004$ (Figure S2).

	I135	I135V	I135L	I135T
Log BL50(nM)	4.07	4.15	5.06	6.64
Log LL50 (nM)	0.11	0.37	0.82	0.94
Log LL50atBL50 (nM)	0.61	0.64	1.03	1.72
kE = Lysis at LL50atBL50 for I135	65.48	64.13	18.6	8.99
Δr		0.01	0.42	0.58
ΔB		0.02	0.21	0.54

Table S1. Estimating Δr and ΔB from Kawashima et al [28] for the three mutations in HLA-B*51 restricted epitope TAFTIPSI (RT 128135) that showed detectable binding to HLA-B*51.

	N260D	N260E
Log BL50(nM)	0.89	2.01
Log LL50 (nM)	-0.3	1.63
Log LL50atBL50	-0.3	1.45
kE = Lysis at LL50atBL50 for N260D	1427.33	73.94
Δr		0.72
ΔB		0.30

Table S2. Estimating Δr and ΔB from Matthews et al [29] for HLA-B*3501 restricted epitope NPPIPVGDIY (Gag 253- 262) for one mutation.
